# Myocardial disease and ventricular arrhythmia in Marfan syndrome: a prospective study

**DOI:** 10.1186/s13023-020-01581-8

**Published:** 2020-10-23

**Authors:** Laura Muiño-Mosquera, Hans De Wilde, Daniel Devos, Danilo Babin, Luc Jordaens, Anthony Demolder, Katya De Groote, Daniel De Wolf, Julie De Backer

**Affiliations:** 1 Centre for Medical Genetics, Ghent University Hospital, Ghent, Belgium; 2 Division of Pediatric Cardiology, Department of Pediatrics, Ghent University Hospital, C. Heymanslaan 10, 9000 Ghent, Belgium; 3 Department of Cardiology, Ghent University Hospital, Ghent, Belgium; 4 Department of Radiology, Ghent University Hospital, Ghent, Belgium; 5 Imec-TELIN-IPE, Ghent University, Ghent, Belgium

**Keywords:** Marfan syndrome, Arrhythmia, Heart failure, Myocardial disease

## Abstract

**Background:**

Aortic root dilatation and—dissection and mitral valve prolapse are established cardiovascular manifestations in Marfan syndrome (MFS). Heart failure and arrhythmic sudden cardiac death have emerged as additional causes of morbidity and mortality.

**Methods:**

To characterize myocardial dysfunction and arrhythmia in MFS we conducted a prospective longitudinal case–control study including 86 patients with MFS (55.8% women, mean age 36.3 yr—range 13–70 yr–) and 40 age—and sex-matched healthy controls. Cardiac ultrasound, resting and ambulatory ECG (AECG) and NT-proBNP measurements were performed in all subjects at baseline. Additionally, patients with MFS underwent 2 extra evaluations during 30 ± 7 months follow-up. To study primary versus secondary myocardial involvement, patients with MFS were divided in 2 groups: without previous surgery and normal/mild valvular function (MFS-1; N = 55) and with previous surgery or valvular dysfunction (MFS-2; N = 31).

**Results:**

Compared to controls, patients in MFS-1 showed mild myocardial disease reflected in a larger left ventricular end-diastolic diameter (LVEDD), lower TAPSE and higher amount of (supra) ventricular extrasystoles [(S)VES]. Patients in MFS-2 were more severely affected. Seven patients (five in MFS-2) presented decreased LV ejection fraction. Twenty patients (twelve in MFS-2) had non-sustained ventricular tachycardia (NSVT) in at least one AECG. Larger LVEDD and higher amount of VES were independently associated with NSVT.

**Conclusion:**

Our study shows mild but significant myocardial involvement in patients with MFS. Patients with previous surgery or valvular dysfunction are more severely affected. Evaluation of myocardial function with echocardiography and AECG should be considered in all patients with MFS, especially in those with valvular disease and a history of cardiac surgery.

## Introduction

Marfan syndrome (MFS) (OMIM #154700, ORPHA #284963) is an inherited connective tissue disorder caused by pathogenic variants in the fibrillin-1 gene (*FBN1*), encoding for the extracellular matrix protein, fibrillin-1 [1]. Although aortic root dilatation and—dissection and mitral valve prolapse (MVP) are the most common and best studied cardiovascular manifestations in MFS, heart failure and sudden cardiac death, presumably secondary to ventricular arrhythmia, seem to be additional causes of morbidity and mortality to consider in at least some of these patients [2, 3].

Ventricular myocardial dysfunction in MFS is usually mild and affects 7–68% of the patients, depending on the studied cohort, the technique used and the definition of ventricular dysfunction [4–10]. End-stage heart failure is less common and occurs only in a minority of patients [3, 11, 12]. Left ventricular enlargement and myocardial dysfunction in MFS can result from valvular disease, but primary biventricular myocardial involvement in patients with MFS without valvular pathology has been shown by several independent groups [5–10]. Furthermore, mild myocardial impairment and abnormal myocardial signaling have been shown in 2 different murine models of MFS (*fbn1*^mgR/mgR^ and *fbn1 *^*C1039G*/+^) [13, 14]. Using one of these models (*fbn1 *^*C1039G/*+^), Rouff et al. [15] further showed that hemodynamic overload by TAC ligation (transverse aortic constriction), was less well tolerated in MFS mice than in wild-type littermates. This finding supports the idea that secondary hemodynamic overload in a myocardium primarily predisposed to disease might cause more damage.

Next to ventricular enlargement or dysfunction, myocardial disease might manifest as ventricular arrhythmia. The prevalence of sustained ventricular tachycardia (VT) and sudden cardiac death (SCD) in MFS is approximately 10% and 4% respectively [2, 4, 16]. An enlarged left ventricle [4, 16, 17] and high levels of NT-proBNP [2, 16] seem to be the most consistent features related to ventricular arrhythmia. Isolated MVP without regurgitation, although associated with ventricular arrhythmia and SCD in non-MFS populations [18], has not shown a consistent relation with arrhythmia in MFS [2, 4, 16, 17].

Genotype–phenotype correlations have been studied by a few groups. Aalberts et al. [19] found a higher prevalence of left ventricular dilatation in non-missense *FBN1* variant carriers and Aydin et al. [16] found that patients carrying a *FBN1* missense variant were more likely to present ventricular ectopy. Given the known relation between left ventricular dimension and the presence of ventricular ectopy, these results seem contradictory and warrant further study.

To investigate myocardial disease in patients with MFS, we conducted a prospective longitudinal case–control study. The aims of the study were: (1) Compare the prevalence of myocardial dysfunction and arrythmia between patients with MFS and sex- and age-matched healthy controls. (2) Distinguish primary versus secondary myocardial involvement. (3) Identify factors predisposing to myocardial disease and (4) Study genotype–phenotype correlations.

## Materials and methods

### Subjects

**Fig. 1:**
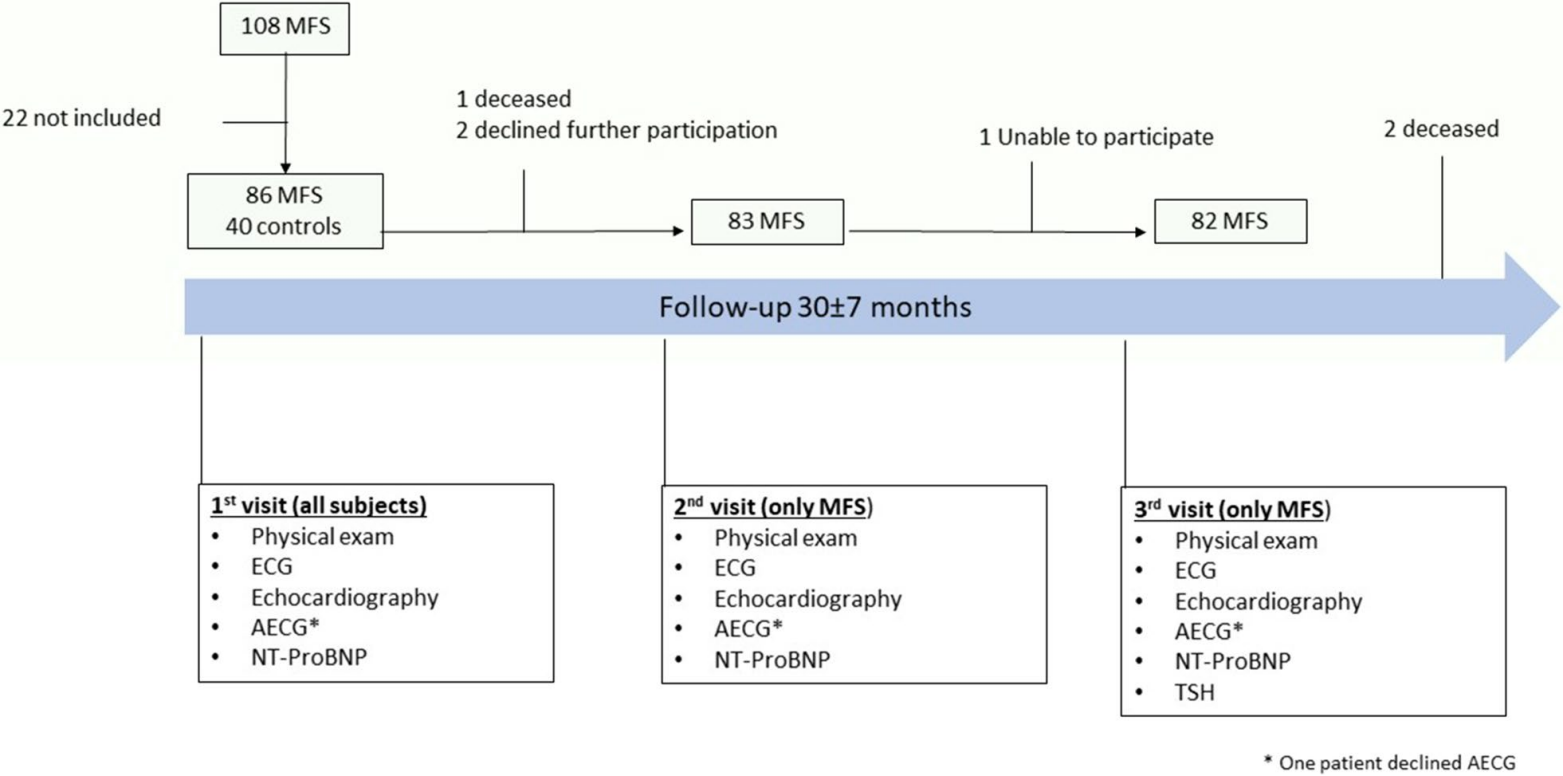
Inclusion procedure and investigations at baseline and during follow-up. Eighty-six patients with MFS and 40 age- and sex matched controls were included in the study. At baseline all subjects underwent physical examination, resting and ambulatory ECG, cardiac ultrasound and dosing of NT-proBNP. In patients with MFS these investigations were repeated twice, in a mean period of 30 ± 7 months and TSH level was determined at the end of the study. Additionally, a subset of 45 patients underwent cardiac MRI with angiography and measurement of PWV. Eighty-two patients completed all 3 visits. Three patients died during study, 2 of them after completing the 3rd visit. Two patients declined further participation after the 1st visit and 1 patient could not attend the last visit. *AECG* ambulatory ECG, *ECG* electrocardiogram, *MFS* Marfan syndrome, *MRI* magnetic resonance image

Patients with a clinical diagnosis of MFS according to the revised Ghent nosology [1] older than 12 yr and in whom a (likely) pathogenic *FBN1* gene variant was confirmed, were asked to participate in the study. Of the 108 patients evaluated in our institution between January 2015 and June 2016 and fulfilling the inclusion criteria, 86 agreed to participate in this longitudinal study. Six patients were excluded due to psychosocial problems (N = 5) or residency outside Belgium (N = 1), 11 patients declined participation, 4 patients were not included because of (planned) pregnancy and 1 patient because of a medical history of heart transplantation. Forty age- and sex-matched healthy volunteers were also recruited (Fig. 1). To participate in the study, controls could not have a (family) history of bicuspid aortic valve, thoracic aortic disease or cardiomyopathy.

In all control and MFS subjects, personal medical and family history, medication use and conventional cardiovascular risk factors (self-reported diabetes, hypercholesterolemia and smoking habits) were documented. Anthropometric data and blood pressure after 10 min resting were assessed. A 12-lead electrocardiogram (ECG), a 24-h ambulatory ECG (AECG) and a standard cardiac ultrasound were performed. NT-proBNP was measured at baseline. Additionally, patients with MFS were followed for a mean duration of 30 ± 7 months. During follow-up, they underwent annual ECG and 24 h AECG recording, cardiac ultrasound and measurement of NT-proBNP. Since thyroid function is associated with higher risk of arrythmia [20, 21], levels of TSH were measured in all patients at the end of the study to rule out (sub)clinical thyroid disease.

To evaluate primary versus secondary myocardial disease, patients with MFS were divided in two groups: the 1st group without medical history of cardiovascular surgery [aortic root replacement (AoRR) or isolated mitral valve surgery] and without moderate to severe mitral or aortic regurgitation. This group is further referred in the text as MFS-1 (N = 55). The 2nd group of patients with either cardiovascular surgery or with valvular disease (or both), is further referred in the text as MFS-2 (N = 31).

### Study procedures

Cardiac ultrasound was performed with a Vivid S60N^®^, GE Healthcare, equipped with a 5S probe. Aortic diameters, valvular function as well as cardiac chamber dimensions and—function were measured according to the guidelines of the American Society of Echocardiography (ASE) and the European Association of Cardiovascular Imaging (EACVI) [22, 23]. Data were stored and analyzed offline using an ultrasound workstation (EchoPAC, GE Healthcare, version 201). For the continuous variables, an average of 3 consecutive measurements was calculated. Aortic and mitral valve regurgitation were graded into mild, moderate or severe and mitral valve prolapse was considered if there was a superior mitral leaflet displacement of more than 2 mm in systole. Z-scores of the aortic sinus and proximal ascending aorta were calculated according to Campens et al. [24]. For evaluation of diastolic function, a combination of pulsed wave and tissue doppler imaging (TDI) was used. LV ejection fraction (EF) was calculated from a 2D image in parasternal long-axis view (LVEF = LVEDD^2^-LVESD^2^/LVEDD^2^*100 + k, where LVEDD = left ventricular end diastolic diameter, LVESD = left ventricular end systolic diameter, k = correction for apical contraction). Systolic dysfunction was defined as LVEF < 55% [23]. Mid-wall fractional shortening (mw-FS), left ventricular mass (LVM) and relative wall thickness (RWT) were also derived from the 2D image in parasternal long-axis using the formulas recommended by the ASE and EACVI [23]. A LVEDD was considered enlarged if indexed for body surface area (BSA)(LVEDDi) was above 30 mm/m^2^ [23]. The tricuspid annular plane systolic excursion (TAPSE) viewed from the four-chamber view was used to evaluate right ventricular function. TAPSE was considered abnormal if ≤ 16 mm.

A routine 12-lead ECG was recorded using an available commercial system (MAC 5500 HD, GE Healthcare). Regular measurements of P-wave width and height, P-wave axis, PR-interval, QRS width and axis, and QTc interval using Bazett’s formula were calculated. The U-wave was included in the measurement of the QTc interval if > 50% of the T-wave height. A standard AECG was performed and analyzed using 2 semi-automatic software packages (Philips DigiTrack XT^®^, Philips and Trillium Platinum TM^®^, Forest Medical). Minimum, maximum and average heart rate, number of supraventricular—(SVES) and ventricular extrasystoles (VES) were recorded. Atrial runs and non-sustained ventricular tachycardia (NSVT) were defined as 3 or more consecutive atrial or ventricular beats. Sustained ventricular tachycardia (VT) was defined if VT lasted 30 s or more. Ventricular ectopy (VE) was defined if more than 10 VES/h. Heart rate variability (HRV) was studied using the standard deviation of the normal-to-normal interval (SDNN) and the square root of the mean squared difference of successive normal-to-normal intervals (RMSDD).

### Classification of the genetic variants

To study genotype–phenotype correlations, variants in the *FBN1* gene were classified according to their effect on the DNA structure as missense (single nucleotide change), in-frame (indels not causing alteration in the reading frame), frameshift (indels causing an alteration in the reading frame), nonsense (single nucleotide change causing a premature stop-codon) and splice-site variants (indels or single nucleotide change in a place where splicing occurs).

Variants were also classified according to the expected effect at the protein level [25]. Frameshift and nonsense variants not affecting exon 65 or the last 50 nucleotides of exon 64 were considered to have an haploinsufficient (HI) effect, leading to the production of a reduced amount of normal fibrillin-1 (derived from the non-mutated allele). The other frameshift and nonsense variants and all missense variants were considered to have a dominant negative (DN) effect, leading to a shorter or a structurally abnormal but stable protein. These predictions were confirmed by the Mutation Taster software [26]. To classify the effect of the splice-site variants we used the Human Splicing Finder Software [27]. Splice-site variants causing a change in the reading frame were considered as HI, while variants affecting splicing but not causing a change in the reading frame were considered as DN.

Furthermore, we considered variants affecting exon 24–32 separately because these have been previously associated with higher ventricular ectopy [16].

### Statistical analysis

Statistical analysis was performed using IBM SPSS Statistics 25 package (SPSS Inc., Chicago, IL, USA).

Continuous variables are expressed as mean and standard deviation or as median and interquartile range (IQR). Categoric variables are expressed as absolute value and percentage.

The Kolmogorov–Smirnov test was used to determine normality. Continuous variables were analyzed either using the unpaired sample *t*-test and the Mann–Whitney-U if two groups were compared or the ANOVA and Kruskal–Wallis if more than 2 groups were compared. For the categorical variables Chi-square test or the Fisher exact test were used. Variables were adjusted for confounders using either linear regression for continuous variables or logistic regression for categoric variables. Those variables with a *p*-value < 0.2 were considered as possible confounders. Since NT-ProBNP and the amount of ventricular extrasystoles (VES) in 24 h were extremely skewed, a logarithmic transformation was performed to include them in the multivariable analysis.

### Ethical issues

The study was approved by the local Independent Ethics Committee and the Institutional Review Board (IRB) of our hospital. All subjects participating in the study gave written informed consent.

## Results

### Baseline characteristics and comparison between Marfan syndrome patients and control subjects

Eighty-six patients with MFS (55.8% female, 36.3 ± 14.3 yr—range 13–70 yr–) and 40 age- and sex-matched controls (52.5% female, 37.9 ± 14.4 yr—range 16–69 yr) were included in the study. One of the study patients declined AECG (Fig. 1).

Twenty-four patients had undergone cardiovascular surgery at baseline (21 AoRR, 2 mitral valve surgery alone and 1 patient both). Eleven patients had moderate to severe valvular disease (5 mitral valve regurgitation, 4 aortic valve regurgitation and 1 both) of whom 7 had not undergone cardiovascular surgery. These 31 patients formed the MFS-2 group. Eight patients had previously been treated or were currently under treatment for atrial arrhythmia (4 atrial fibrillation and 4 with another type of supraventricular tachycardia) and 3 patients had been treated for symptomatic ventricular ectopia (2 medical treatment and 1 ablation). As shown in Table 1 patients in the MFS-1 and MFS-2 groups did not differ significantly except for in the incidence of atrial events in their medical history. Afib in particular was more frequent in the MFS-2 group. In the control group, except for one woman of 69 years old who had undergone coiling for a cerebral aneurysm, no one had a significant medical history. Two controls had smoked in the past, 2 were treated for arterial hypertension and 7 reported to have hyperlipidemia.

**Table 1:** Baseline characteristics of the patients with Marfan syndrome

Parameter	MFS-1 (N = 55)	MFS_2 (N = 31)	*p* value
Female (%)	31 (56.4)	17 (54.8)	0.891
Age	35.07 ± 14.7	38.5 ± 13.7	0.295
FBN1 variant type (%)			0.296
Missense	29 (53.7)	13 (41.9)	
Frameshift	13 (24.1)	7 (35)	
Nonsense	9 (16.7)	6 (19.4)	
Splice-site	2 (28.6)	5 (71.4)	
De novo (%)	17 (30.9)	8 (26.7)	0.682
Systemic score^a^	8.02 ± 3.3	8.7 ± 3	0.401
EL (%)	25 (48.1)	21 (70)	0.149
AoRR (%)	0	22 (71)	n.a
Valve sparing (%)		16 (72.7)	
Valve replacement (%)		6 (27.3)	
MVP (%)			0.393
Bulging	17 (52.8)	7 (23.3)	
Prolapse	8 (15.1)	8 (26.7)	
MV surgery (%)	0	3 (9.3)	n.a
Valvuloplasty and ring (%)		1	
Bioprosthesis (%)		1	
Mechanical valve (%)		1	
Atrial arrhythmia (%)^b^			0.016*
Afib (%)	0	4 (12.9)	
Other SVT (%)	2 (3.6)	2 5 (6.4)	
Symp. VE (%)^b^	1	2	0.261
Treatment			0.206
None (%)	16 (29.1)	3 (9.7)	
BB alone (%)	19 (34.5)	16 (51.6)	
ARB alone (%)	6 (10.9)	3 (9.7)	
ACEi alone (%)	1 (1.8)	0	
BB + ARB (%)	13 (23.6)	8 (25.8)	
BB + ACEi (%)	0	1 (3.2)	
BMI ≥ 25 kg/m^2^ (%)	13 (23.6)	7 (23.3)	0.365
Smoking (%)			0.979
Never	38 (69.1)	22 (71)	
Ex-smoker	11 (20)	6 (19.4)	
Current	6 (10.9)	3 (9.7)	
AHT (%)	6 (10.9)	2 (6.5)	0.494
Hyperlipidaemia (%)	5 (9.1)	3 (9.7)	0.844
Diabetes (%)	3 (5.5)	0	0.186

*ACEi* angiotensin converting enzyme inhibitor, *Afib* atrial fibrillation, *AoRR* aortic root replacement, *ARB* angiotensin II receptor blocker antagonist, *BB* beta-blocker, *BMI* body mass index, *EL* ectopia lentis, *HTA* arterial hypertension, *MV* mitral valve, *MVP* mitral valve prolapse, *SVT* supraventricular tachycardia, *VE* ventricular ectopy

^a^Marfan systemic score is a scoring system which takes into consideration several characteristic features of MFS and assigns each of them a value between 1 and 3, 3 being the most specific for the disease. A score of ≥ 7 is considered abnormal and in combination with aortic disease and/or ectopia lentis is diagnostic of MFS

^b^Patients reporting palpitations in the past were not included. Only confirmed atrial and ventricular ectopy for which treatment was implemented were considered

As shown in Table 2, while 7 (8.1%) of the patients with MFS had mildly decreased LVEF (5 in the MFS-2 group), decreased LVEF was no present in control subjects.

**Table 2:** Comparison between control subjects and patients with Marfan syndrome with and without valvular disease and/or cardiovascular surgery

	Control subjects	Patients with Marfan syndrome		*p* value	*p* value
	N = 40	No surgery	Surgery	Control versus MFS-1	MFS-1 versus MFS-2
		No valvular disease	Valvular disease		
		N = 55 (MFS-1)	N = 31 (MFS-2)		
Female (%)	22 (55)	31 (56.4)	17 (54.8)	0.895	0.891
Age (%)	37.9 ± 14.4	35.1 ± 14.7	38.5 ± 13.7	0.370	0.295
Height (cm)	173 ± 10	183.2 ± 11	183.9 ± 9.5	< 0.001	0.771
Weight (kg)	68.3 ± 9.2	74.2 ± 17.9	74.1 ± 18.3	0.041	0.987
BSA (m^2^)	1.8 ± 0.1	1.9 ± 0.2	1.9 ± 0.2	0.001	0.923
SBP (mmHg)	121.1 ± 12.7	121 ± 12.6	127.3 ± 15.9	0.899	0.049
DBP (mmHg)	73.7 ± 9.6	71.6 ± 70	67 ± 10.1	0.361	0.067
BB Use (%)^a^	0 (0)	32 (58.2)	25 (80.6)	n.a	0.034
Ao sinus (mm)	30.5 ± 2.9	39.8 ± 5.1	41.1 ± 6.6^b^	< 0.001	0.498
z-score sinus	− 0.8 ± 0.7	2.5 ± 1.4	3.3 ± 1.1^b^	< 0.001	0.094
LVEDDi (mm/m^2^)	24.7 ± 2.4	24.8 ± 3.4	26.7 ± 3.5	0.795	0.020
LVEDDi ≥ 30 mm/m^2^ (%)	0 (0)	5 (9.1)	4 (12.9)	0.050	0.717
iLVM	86.9 ± 20.2	81 ± 34.7	86.2 ± 23.04	0.331	0.486
LVEF (%)	68.3 ± 7.2	66 ± 7.2	62.5 ± 7.6	0.131	0.034
LVEF < 55%	0 (0)	2 (3.6)	5 (16.1)	0.507	0.093
mw-FS (%)	21 ± 7	21 ± 5	16 ± 6	0.778	0.004
RWT	0.45 (0.40–0.50)	0.37 (0.32–0.45)	0.33 (0.29–0.40)	< 0.001	0.060
E/A	1.5 (1.2–2)	1.6 (1.3–2)	1.6 (1.2–2.1)	0.351	0.975
E/E’	6.4 (5.1–7.6)	7.8 (6.6–9.3)	8.6 (7–12.8)	< 0.001	0.051
LAVi (ml/m^2^)	21.2 ± 5.5	21.4 ± 1.3	26.4 ± 11.3	0.945	0.038
MVP (%)^c^	1 (2.5)	8 (14.5)	8 (26.7)	0.045	0.185
TAPSE (mm)	24.5 ± 2.8	22.1 ± 4.3	19.5 ± 3.8	0.003	0.010
TAPSE ≤ 16 mm (%)	0 (0)	3 (6.4)	6 (20.7)	0.250	0.077
NT-ProBNP (pg/ml)	30 (19–44.5)	53.5 (30–74.2)	129 (82–235.2)	0.001	< 0.001
Min HR (bpm)	50.5 (44–55.5)	45 (41.5–49)	48 (44–55)	0.004	0.026
Average HR (bpm)	74.2 ± 8.3	64 (56.5–71.5)	67 (58.5–73)	< 0.001	0.205
QRS width (ms)	80 (80–90)	96 (86–104)	98 (86–106)	< 0.001	0.639
QTc (ms)	380.2 ± 24.5	414 (388–433.5)	426 (407–445)	< 0.001	0.028
QTc > 460 ms (%)	0 (0)	0 (0)	4 (12.9)	n.a	n.a
SVES/24 h	2 (0.25–4.7)	7 (2–37.5)	17 (1–56)	< 0.001	0.560
Atrial runs (%)	2 (5)	14 (26.4)	12 (41.4)	0.015	0.318
VES/24 h	0 (0–5.7)	6 (1–69.5)	14 (1.5–373.5)	< 0.001	0.312
VE (%)	5 (12.5)	14 (26.4)	13 (41.9)	0.099	0.090
Vent couplets (%)	2 (5)	9 (17)	8 (25.8)	0.077	0.270
NSVT (%)	0 (0)	5 (9.1)	5 (17.2)	0.050	0.273
SDNN (ms)	147 (116–185.2)	185 (156.2–219.2)	132 (95.4–191)	0.001	0.003
RMSDD (ms)	53 (36.2–84)	82.5 (66.2–82.5)	59.3 (42.7–112)	< 0.001	0.040

Values are given as mean ± SD, median (IQR) or number (%)

*Ao* aortic, *BB* beta-blocker, *BSA* body surface area, *DBP* diastolic blood pressure, *Dec time* deceleration time, *HR* heart rate, *LAVi* left atrium volume index, *LVEDDi* left ventricular end diastolic diameter index, *LVEF* left ventricular ejection fraction, *MFS* Marfan syndrome, *MVP* mitral valve prolapse, *mw-FS* mid-wall fractional shortening, *NSVT* non-sustained ventricular, *RMSDD* mean squared difference of successive NN intervals, *RWT* relative wall thickness, *SBP* systolic blood pressure, *SDNN* standard deviation of the NN interval, *SVES* supraventricular extrasystoles, *VES* ventricular extrasystoles, *TAPSE* tricuspid annular plane systolic excursion, *VE* ventricular ectopy

^a^Beta-blocker alone or in combination

^b^Only those patients with valvular pathology without aortic root replacement are considered for the mean value of the sinus and the z-score

^c^Only those patients with true mitral valve prolapse considered here. Those with mitral valve bulging were not included in this calculation

Demographic characteristics in the group with decreased LVEF were similar to the rest of the MFS cohort (Additional file 1: Table S1). Median LVEF in this group was 53.1% (IQR 47.1–53.7%). As expected, LVEDDi and LVESDi were significantly higher in patients with decreased LVEF (28.2 ± 3.5 mm/m^2^ versus 25.3 ± 3.5 mm/m^2^, *p* = 0.038 and 21.7 ± 2.6 mm/m^2^ versus 16.6 ± 2.7 mm/m^2^, *p* < 0.001, respectively). Nine patients (10.5%) had a TAPSE value ≤ 16 mm (6 in the MFS-2 group).

To assess whether myocardial involvement in MFS has a primary component, we compared control subjects with those MFS-1 patients without previous surgery or valvular disease. As shown in Table 2, mild biventricular myocardial involvement in MFS-1 was evidenced by significantly higher NT-proBNP, left ventricular dimension, E/Em ratio and lower RWT and TAPSE. Furthermore, MFS-1 patients showed longer QRS-duration and QTc time at rest ECG. NSVT only occurred in patients with MFS. SDNN and RMSDD were significantly higher in MFS-1 even after adjusting for beta-blocker use (*p* = 0.008 and *p* = 0.027, respectively).

In comparison to MFS-1 patients, MFS-2 patients showed significantly larger left ventricular dimensions, lower left ventricular ejection fraction, mw-FS and TAPSE, higher NT-proBNP and longer QTc time. QTc time was abnormal (> 460 ms) in 4 patients in the MFS-2 group. Systolic blood pressure was also slightly but significantly higher in MFS-2 patients (Table 2). SDNN and RMSDD were significantly lower in MFS-2 and similar to the control population.

### Events during follow-up and AECG characteristics in subsequent examinations

All patients except 4 completed the study (one patient died and 2 patients declined further participation after visit 1 and one patient could not attend the last visit—Fig. 1).

During a follow-up period of 30 ± 7 months 3 patients died from (suspected) aortic dissection or rupture (2 type A and 1 type B). Two of them died just after completing the 3rd visit (Fig. 1). Four other patients survived a dissection (1 coronary dissection, 1 type B dissection and 2 type A dissections). Prophylactic AoRR was performed in 6 patients and aortic valve replacement with mechanical prosthesis in an additional patient who had previous AoRR.

As shown in Additional file 1: Table S2, LVEDD and LVEF remained stable during the study follow-up. There were no patients showing heart failure symptoms during follow-up. The overall amount of VES on 24 h AECG in patients with MFS at baseline was low (7.5 VES/24 h IQR 1–98). However, 20 patients (23.3%) showed NSVT on one or more of the 24 h AECG (10 at the 1st exam, 10 additionally during the 2 consecutive exams). 80% of these patients was under treatment with a beta-blocker (Table 3). Only one patient in this group, showed sustained VT during follow-up. He was 30 years old at the time of the first sustained VT episode and had had AoRR and MV surgery 4 months earlier. An ICD was implanted and showed recurrent episodes of VT under beta-blocker therapy. VT was only controlled with amiodarone therapy. No patients in the study developed SCD or arrhythmogenic syncope during the study period.

**Table 3:** Comparison between patients with and without non-sustained ventricular tachycardia

	Non-sustained ventricular tachycardia		
	Present (n = 20)	Absent (n = 65^b^)	*p* value
Female (%)	10 (50)	37 (59.7)	0.604
Age (yrs)	40.3 ± 15.7	35.2 ± 13.8	0.170
BSA (kg/m^2^)	21.3 (19–25.5)	21.4 (18.6–24.8)	0.821
SBP (mmHg)	124.3 ± 12.8	122.1 ± 14.5	0.549
DBP (mmHg)	67.7 ± 5.7	70.1 ± 10.9	0.365
BB use (%)	16 (80)	39 (62.9)	0.157
MFS systemic score^a^	8 (4–11)	8 (6–10)	0.657
MFS group			*0.008*
MFS-1 (N = 55) (%)	8 (40)	47 (72.3)	
MFS-2 (N = 30^b^) (%)	12 (60)	18 (27.7)	
Cardiac ultrasound			
Ao sinus (mm)	39.9 ± 4.3	40.1 ± 5.6	0.906
MVP (%)	7 (35)	9 (15.3)	0.058
LVEDDi (mm/m^2^)	27.9 ± 3.6	24.8 ± 3.2	< *0.001*
LVESDi (mm/m^2^)	18.4 ± 3	16.7 ± 2.7	*0.028*
LVEF (%)	63.6 ± 8.2	65.2 ± 7.4	0.419
Mw-FS (%)	21 ± 9	19 ± 6	0.344
RWT	0.36 (0.31–0.44)	0.35 (0.29–0.40)	0.354
RVOTi (mm/m^2^)	15.9 ± 2.4	15.4 ± 2.7	0.476
TAPSE (mm)	20.8 ± 3.7	21.7 ± 4.2	0.448
LAVi (mm/m^2^)	24.2 (14–29.2)	21.2 (15.2–28.6)	0.854
E/A ratio	1.6 ± 0.6	1.7 ± 0.6	0.723
E/Em ratio	9.5 (7.1–11.7)	7.8 (6.6–9.7)	0.100
Serologic test			
NT-ProBNP (pg/ml)	112 (78.5–216.5)	60 (31–129)	*0.017*
Ambulatory ECG			
Min HR (bpm)	47 (42–51)	46 (42.2–50)	0.792
Average HR (bpm)	67 (58–72)	65.5 (57.2–72.7)	0.991
Max HR (bpm)	122 (100–133)	120.5 (101–142)	0.684
QRS width (ms)	100 (90.5–109.5)	96 (82.5–101.5)	0.075
Qtc time (ms)	426 (403–443.2)	413 (385.2–432)	0.199
VES/24 h	345 (9–3727)	4 (1–35)	*0.001*
SDNN (ms)	177 (126.1–264.6)	176 (140–203.5)	0.620
RMSDD (ms)	74 (58–159.9)	79.5 (57.9–109)	0.790

Values are given as mean ± SD, median (IQR) or number (%)

*Ao* aortic, *AoRR* aortic root replacement, *AR* aortic regurgitation, *BB* beta-blocker, *BSA* body surface area, *DBP* diastolic blood pressure, *ECG* electrocardiogram, *HR* heart rate, *LAVi* left atrium volume index, *LVEDDi* left ventricular end diastolic diameter index, *LVESDi* left ventricular end systolic diameter index, *LVEF* left ventricular ejection fraction, *LVMi* left ventricular mass index, *MFS* marfan syndrome, *MR* mitral regurgitation, *MV* mitral valve, *MVP* mitral valve prolapse, *mw-FS* mid-wall fractional shortening, *NSVT* non-sustained ventricular, *RAVi* right atrium volume index, *RMSDD* mean squared difference of successive NN intervals, *RVOT* right ventricular outflow track, *RWT* relative wall thickness, *SBP* systolic blood pressure, *SDNN* standard deviation of the NN interval, *SVES* supraventricular extrasystoles, *TAPSE* tricuspid, annular, plane systolic excursion, *VES* ventricular extrasystoles

^a^MFS systemic score is a scoring system which takes into consideration several characteristic features of MFS and assigns each of them a value between 1 and 3, 3 being the most specific for the disease. A score of ≥ 7 is considered abnormal and in combination with aortic disease and/or ectopia lentis is diagnostic of MFS

^b^One patient declined 24 h AECG

**Fig. 2:**
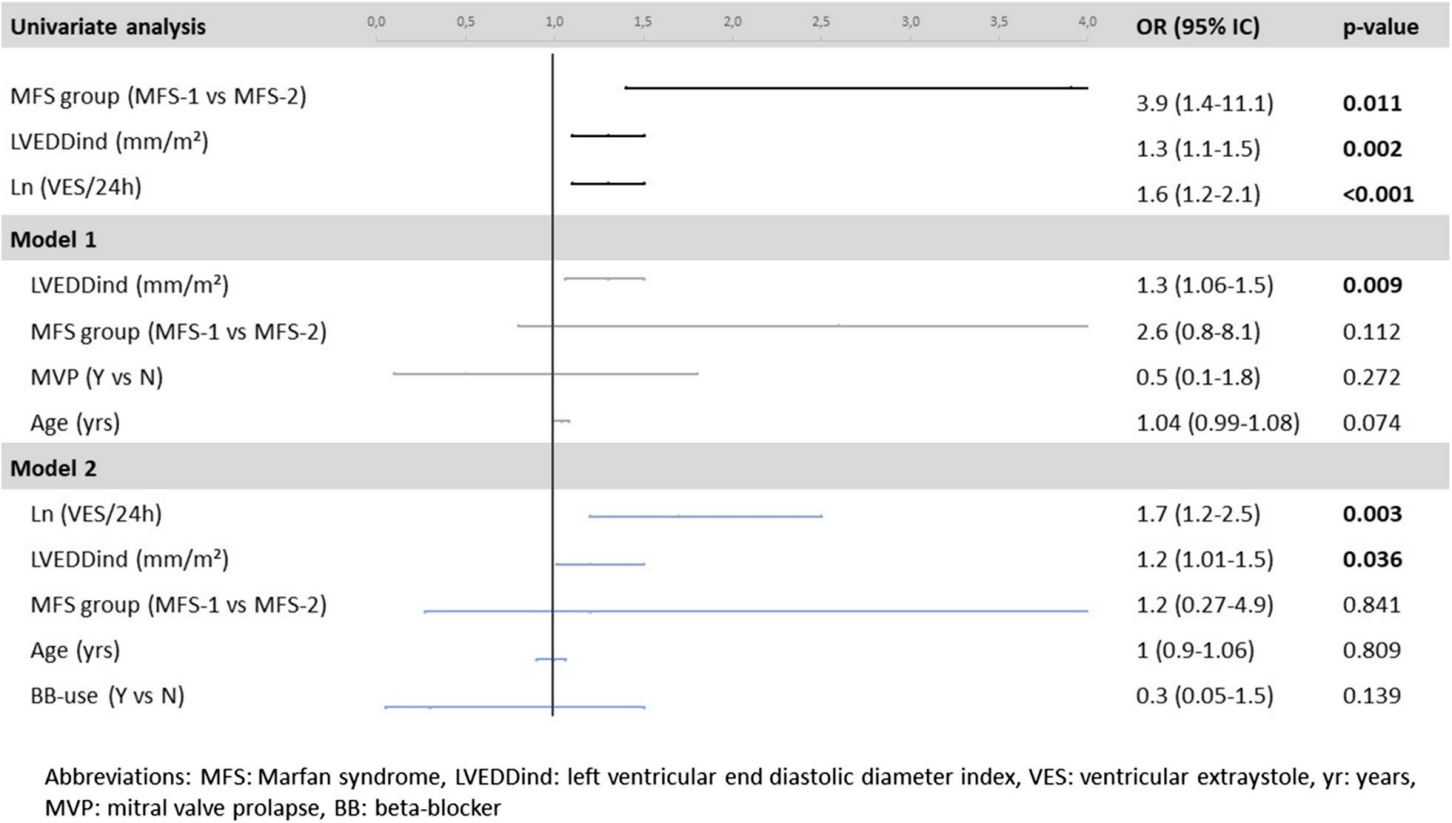
Multivariate analysis to identify independent associations with NSVT in patients with Marfan syndrome. Multivariate analysis shows that higher left ventricular dimension and higher amount of VES in the AECG are the only independent factors of the presence of NSVT. *ln* logarithm, *LVEDDind* left ventricular end diastolic diameter index, *MFS* Marfan syndrome, *ln* logarithm, *yr* year, *VES* ventricular extrasystoles

To identify factors associated with NSVT we compared the 20 patients having NSVT with the rest of the MFS cohort. Univariate analysis showed that LVEDDi, LVESDi, NT-proBNP and the amount of VES/24 h were significantly higher in the group with NSVT (Table 3). Furthermore, NSVT during follow-up occurred more frequently in the MFS-2 group than in the MFS-1 group (40% versus 14.5%, *p* = 0.008). In multivariate analysis, however, only LVEDDi and VES/24 h were independently associated with NSVT (Fig. 2). These 2 variables were associated with NSVT independently from each other.

Other factors such as the presence of MVP or increased E/Em ratio tended to be higher in the group with NSVT but was not statistically significant. We could not identify variables on the resting ECG or parameters of HRV associated with the presence of NSVT.

At baseline patients in the MFS-2 group had significantly higher amounts of atrial events (SVT or Afib) in their medical history compared to patients in the MFS-1 group. Although baseline AECG showed higher amounts of supraventricular extrasystoles and atrial runs in the MFS-1 group compared to controls, the prevalence of these events was similar between both MFS groups, as shown in Table 1. During follow-up 4 patients presented Afib, 2 in the MFS-1 group and 2 in the MFS-2 group. A description of clinical characteristics of these patients can be found in the Additional file 1: Table S3’.

Seven patients were under treatment for thyroid disease during the study period (6 women for hypothyroidism with levothyroxine and 1 male for hyperthyroidism with thiamazole). Within the patients with hypothyroidism, one had NSVT during FU and one a history of Afib. All other patients, except for 1 with slightly elevated TSH (5.4 mU/l), had normal values (reference value in our institution 0.4–4 mU/l). This patient showed no arrhythmic events on his three AECGs.

### Genotype–phenotype correlations

As shown in Table 4, the presence of NSVT was not associated with a specific type of *FBN1* variant. We observed, however, that those patients carrying a missense variant tended to have less arrhythmia. Four out of the 7 patients (57.1%) with decreased LVEF carried a frameshift variant. This was significantly higher than in the group with normal LVEF (20.5%, *p* = 0.050). No other genotype–phenotype correlations were found.

**Table 4:** Comparison of the genotype between patients with and without arrhythmia and with normal or decreased ejection fraction

	Non-sustained ventricular tachycardia			Left-ventricular ejection fraction		
	Present (n = 20)	Absent (n = 64)^a^	*p* value	< 55% (n = 7)	≥ 55% (n = 77)	*p* value
Type of variant						
Missense (%)	7 (35)	35 (54.7)	0.100	1 (14.3)	41 (52.6)	0.058
Inframe (%)	1 (5)	0 (0)	0.238	0 (0)	1 (10.3)	0.918
Frameshift (%)	5 (25)	14 (21.9)	0.494	4 (57.1)	16 (20.5)	*0.050*
Nonsense (%)	4 (20)	11 (17.2)	0.747	0 (0)	15 (19.2)	0.243
Splice-site (%)	3 (15)	4 (6.3)	0.349	2 (28.6)	5 (6.4)	0.100
Effect on the protein						
Haploinsufficiency (%)	9 (45)	24 (37.5)	0.128	4 (57.1)	30 (38.5)	0.283
Localization: exon						
Exon 24–32 (%)	2 (10)	8 (12.5)	0.559	0 (0)	10 (12.8)	0.402

Values are given as number (%)

^a^Variant details were lacking in one patient. Another patient declined ambulatory ECG

## Discussion

In our study, we show mild myocardial involvement in patients with MFS, even in those without valvular disease or previous cardiovascular surgery. Prevalence of NSVT was rather high in MFS, presenting in almost a quarter of the patients either at baseline or during follow-up. Those patients with valvular and/or cardiovascular surgery in the past seemed to have higher risk of myocardial disease. Atrial events were higher in MFS patients than in controls and Afib occurred in 9% of the patients in our cohort.

Mild but significant myocardial dysfunction in patients with MFS has been reported earlier in several independent studies [4–10]. Although we did not study the role of aortic stiffening in myocardial involvement, the fact that we noticed mild myocardial dysfunction and a higher rate of arrhythmic events in MFS-1 patients suggests primary myocardial involvement. Furthermore, the fact that the majority of the patients with reduced ejection fraction were carrying a truncating variant and that we found a similar trend for those patients with NSVT, suggests that there is a genetic predisposition, independent of hemodynamic overload, to develop myocardial disease. These findings are in line with the findings of Aalberts et al. [19] and with the general observation that variants causing a HI effect (like most of the truncating variants) are associated with a more severe cardiovascular phenotype [28–31]. On the other hand we demonstrate that those patients with valvular disease and past history of cardiovascular surgery, have larger left ventricular diameters and lower ejection fraction, indicating that hemodynamic overload (as expected) also plays an important role in the pathophysiology of MFS cardiomyopathy. Patients with NSVT in consecutive AECGs did not show progression of valvular dysfunction but we did observe that more patients in the MFS-2 group showed more NSVT during follow-up. We think that this is more related to the prior aortic surgery than to progression of valvular disease. We did not include a specific control group for MFS-2 (non-Marfan syndrome patients after cardiovascular surgery or valvular disease), therefore we are not able to assess whether the effect of hemodynamic overload is more significant in MFS in comparison to the general population.

So far, 6 studies, ours included, have investigated ventricular arrhythmia in MFS in detail [2, 4, 16, 17, 32]. Additional values of our study are the inclusion of a control population and the availability of serial 24 h AECGs in patients with MFS. Although in control subjects isolated ventricular extrasystoles are a relatively common finding, present in 12.5% of the subjects, NSVT was only present in patients with MFS. Two questions which we have not been able to resolve however, are: (1) does the presence of NSVT predispose to life threatening arrhythmias? And (2) is it necessary to treat NSVT to prevent adverse events? The prognostic value of NSVT in non-MFS patients is very variable [33] and the available evidence to answer this question for patients with MFS is very limited. Yetman et al. [4] found that ventricular ectopy (defined as > 10 VES/h, the presence of couplets or NSVT) was an independent risk factors of SCD. In contrast, although Hoffman et al. [2] and Aydin et al. [16] found a higher amount of NSVT in those patients with ventricular arrhythmic events, these differences were not significant after multivariate analysis. In our study, no patient presented SCD or arrhythmogenic syncope and therefore we were not able to assess the predictive value of NSVT. Nevertheless, the high prevalence of NSVT in our study contrasts with the low prevalence of SCD, suggesting that NSVT might not be the best predictor of SCD. In our cohort, the only patient developing sustained VT, showed progressive ventricular ectopy over the years. After implantation of an ICD, sustained VT kept occurring under beta-blocker treatment. Based on our experience and the fact that in the publication of Yetman et al. [4], 2 of the 3 cases of SCD were under treatment with beta-blocker, we think that treatment of ventricular ectopy with beta-blockers alone seems insufficient, but further study in a larger cohort is warranted.

Another matter of debate is the underlying mechanism responsible for the ventricular ectopy. So far an enlarged LV diameter seems to be the most consistent independent factor associated with an arrhythmic event [4, 16, 17]. Although in the publication of Hoffman et al. LV diameter was not independently associated with SCD and sustained VT, NT-proBNP, a marker of myocardial disease, showed to be a good predictor of these events. NT-proBNP, in our cohort, was also higher in those patients with NSVT, but we could not find a very strong association. The levels of NT-proBNP in our patients with MFS were overall within normal values (68.50 pg/ml, IQR 35.3–149.3) which might explain the lack of association.

A peculiar finding was that the 2 parameters of heart rate variability (SDNN and RMSDD) were increased in patients with MFS without valvular disease or surgery independently of beta-blocker use. Heart rate variability refers to the fluctuation in the beat-to-beat interval of a patient’s heart rate and reflects the autonomic activity of the heart. Lower values of heart rate variability have been related to left ventricular dysfunction and a higher incidence of arrhythmia after myocardial infarction in the general population [34]. In patients with MFS, however, its use has been very limited and actually, higher values of heart rate variability have been associated with cardiovascular risk. Hoffman et al. [2] showed significantly higher RMSSD in those patients reaching the composite end point (ventricular tachycardia or fibrillation, arrhythmogenic syncope or sudden cardiac death) and Mah et al. [17] showed higher values of the triangular index (another parameter of heart rate variability) in those patients with worse aortic outcome. In our study, we could not establish an association between heart rate variability and NSVT or reduced left ventricular function. Further study of autonomic function in patients with MFS could be interesting to gain a better understanding of its relation to the clinical outcome.

### Limitations and future perspectives

One of the most important limitations of our study is the sample size and the low number of events. These have precluded us from answering some important questions. Furthermore, we measured left ventricular function with a solid but low sensitive technique (LVEF and md-FS). Using strain analysis might have yielded a better result. Next to these limitations, our control group was relatively small. Taking a larger control group and performing several AECG might have also detected NSVT in healthy individuals.

The underlying cause of ventricular ectopy should be better elucidated. For example, we did not address myocardial fibrosis as underlying mechanism of ventricular dysfunction and arrhythmia. A small study with cardiac MRI in 35 children with MFS (N = 14) and Loeys-Dietz syndrome (N = 21) showed increased markers of myocardial fibrosis in patients compared to an age-matched control population [35]. Another clue to better understand the pathophysiology of arrhythmia, could be determining the location of the ventricular ectopy. In our study, solely based on the 24 h AECG, it was challenging to accurately determine the ectopic foci. Therefore, electrophysiologic studies in a selected group of patients might be useful. Furthermore, in our study, mitral valve prolapse in itself did not seem to be highly correlated with higher incidence of ventricular arrhythmia, however other studies did find this correlation. It is possible that not so much mitral valve prolapse, but mitral valve annular disjunction (MAD) might be the underlying cause of arrhythmia in MFS, as it has been shown in non-MFS patients with MAD [36]. This correlation should be studied more in-depth.

## Conclusion

Patients with MFS have mild but significant myocardial dysfunction and a higher frequency of ventricular arrhythmia in comparison to healthy subjects. Although the overall amount of VES in patients with MFS was low, almost a quarter of the patients presented NSVT. Based on these facts we recommend surveillance of myocardial function and arrhythmia in all patients with MFS. Those patients with increased LV diameter, decreased LV function, palpitations or additional cardiovascular risk factors including valvular disease and surgery, form a higher risk population.

## Supplementary information

**Additional file 1. Supplemental table 1:** comparison between patients with normal and abnormal LVEF. **Supplemental table 2:** Evolution of left ventricular function during follow-up. **Supplemental table 3:** Clinical characteristics of the patients with atrial fibrillation.

## Data Availability

The datasets generated and analysed during the current study are not publicly available because it could compromise the anonymity and confidentiality of the patient data but are available from the corresponding author on reasonable request.

## Abbreviations

ACEi: Angiotensin converting enzyme inhibitor
AECG: Ambulatory electrocardiogram
Afib: Atrial fibrillation
AoRR: Aortic root replacement
ARB: Angiotensin receptor blocker
ASE: America Society of Echocardiography
BB: Beta-blocker
BMI: Body mass index
BSA: Body surface area
DBP: Diastolic blood pressure
DecTime: Deceleration time
DN: Dominant negative
EACVI: European Association of Cardiovascular Imaging
ECG: Electrocardiogram
EL: Ectopia lentis
FBN1: Fibrillin-1 gene
HI: Haploinsufficient
HR: Heart rate
HRV: Heart rate variability
HTA: Arterial hypertension
LAVi: Indexed left atrial volume
LVEDD: Left ventricular end diastolic diameter
LVEDDi: Indexed left ventricular end-diastolic diameter
LVEF: Left ventricular ejection fractions
LVESD: Left ventricular end-systolic diameter
MAD: Mitral valve disjunction
MFS: Marfan syndrome
MV: Mitral valve
MVP: Mitral valve prolapse
Mw_FS: Mid-wall fractional shortening
NSVT: Non-sustained ventricular tachycardia
RAVi: Indexed right atrial volume
RMSDD: Mean squared difference of successive normal to normal intervals
RVOT: Right ventricular outflow track
RWT: Relative wall thickness
SBP: Systolic blood pressure
SCD: Sudden cardiac death
SDNN: Standard deviation of the normal-to-normal interval
SVES: Supraventricular extrasystoles
TAC ligation: Transverse aortic constriction ligation
TAPSE: Tricuspid annular plane systolic excursion
TDI: Tissue Doppler imaging
VE: Ventricular ectopy
VES: Ventricular extrasystoles
VT: Ventricular tachycardia
Yr: Years
