# Supplementary material for: Myocardial disease and ventricular arrhythmia in Marfan syndrome: a prospective study
Source: Orphanet J Rare Dis. 2020 Oct 23;15:300. doi: 10.1186/s13023-020-01581-8 (PMC7585308; doi:10.1186/s13023-020-01581-8)
Supplement: Supplementary file 1 — Additional file 1. Supplemental table 1: comparison between patients with normal and abnormal LVEF. Supplemental table 2: Evolution of left ventricular function during follow-up. Supplemental table 3: Clinical characteristics of the patients with atrial fibrillation. [file 13023_2020_1581_MOESM1_ESM.docx]

**Supplemental table 1: comparison between patients with normal and abnormal LVEF**

|  | **LVEF<55% (n=7)** | **LVEF>55% (n=79)** | **p-value** |
| --- | --- | --- | --- |
| Age (yr) | 43.9 (30.2-48.6) | 33.8 (21.4-44.9) | 0.279 |
| Sex: female (%) | 4 (57.1) | 44 (55.7) | 0.630 |
| BMI (kg/m²) | 23.3 (21.8-24.8) | 20.1 (18.7-25) | 0.381 |
| BB-use (%) | 4 (57.1) | 53 (67.1) | 0.439 |
| SBP (mmHg) | 124±15.8 | 12.3±14.1 | 0.894 |
| DBP (mmHg) | 71.6±6 | 69.7±11.3 | 0.668 |
| AoRR (%) | 3 (42.9) | 19 (24.1) | 0.250 |
| Ao sinus (mm) | 40 (34-40.75) | 40 (37-44.5) | 0.645 |
| LVEDDi (mm/m²) | 28.2±3.5 | 25.3±3.5 | **0.038*** |
| LVESDi (mm/m²) | 21.7±2.6 | 16.6±2.7 | **<0.001*** |
| LVEF (%) | 53.1 (47.1-53.7) | 65.5 (60.4-72.1) | **<0.001*** |
| Moderate/severe MR (%) | 1 (14.3) | 5 (6.7) | 0.425 |
| Moderate/severe AR (%) | 1 (14.3) | 4 (5.2) | 0.356 |
| MVP (%) | 1 (14.3) | 15 (19.5) | 0.601 |
| NT-proBNP (pg/ml) | 113 (96-129) | 60 (33-155.5) | 0.090 |
| VES/24h | 245 (5-4342) | 6 (1-85) | 0.076 |

*Statistic significant at a level of p<0.05

Abbreviations: LVEF: Left ventricular ejection fraction, BMI: Body mass index, BB: Beta-blocker, SBP: Systolic blood pressure, DBP: Diastolic blood pressure, AoRR: Aortic root replacement, Ao: aortic, LVEDDi: Left ventricular end diastolic diameter index, LVESDi: Left ventricular end systolic diameter index, MR: Mitral regurgitation, AR: Aortic regurgitation, MVP: Mitral valve prolapse, VES: Ventricular extrasystoles.

**Supplemental table 2: Evolution of left ventricular function during follow-up**

|  |  | **Control** | | **MFS-1** | | **MFS-2** | |
| --- | --- | --- | --- | --- | --- | --- | --- |
|  |  |  | **p-value** |  | **p-value** |  | **p-value** |
| **LVEDDi (mm/m²)** | *Baseline* | 24.7±2.4 | 0.083 | 24.8±3.4 | 0.098 | 26.7±3.5 | 0.053 |
|  | *End FU* | 25.4±2.2 |  | 24.3±2.9 |  | 26±3.3 |  |
|  |  |  |  |  |  |  |  |
| **LVEF (%)** | *Baseline* | 68.3±7.2 | 0.607 | 66±7.2 | 0.410 | 62.5±7.6 | 0.368 |
|  | *End FU* | 68.9±6.4 |  | 64.1±6.5 |  | 63.1±6.7 |  |

Abbreviations: LVEDDi: Left ventricular end diastolic diameter index, LVEF: Left ventricular ejection fraction, FU: follow-up, MFS: Marfan syndrome

**Supplemental table 3: Clinical characteristics of the patients with atrial fibrillation**

| **Case** | **Sex** | **Age (yr)** | **BMI (kg/m²)** | **HTAa** | **BB** | **AoRR** | **LAVi (ml/m²)** | **E/Em** | **MVP** | **MR** |
| --- | --- | --- | --- | --- | --- | --- | --- | --- | --- | --- |
| **Afib in medical historyb** | | | | | | | | | | |
| 1 | F | 56 | 25.7 | N | Sotalol | Y | 36 | 12 | N | N |
| 2 | F | 27 | 20.5 | N | Bisoprolol | Y | 41 | n.a | Surgery | Moderate |
| 3 | F | 57 | 21.6 | N | Sotalol | N | 51 | n.a | Surgery | N |
| 4 | F | 30 | 20.4 | N | Metoprolol | N | 25 | n.a | Surgery | N |
| **Afib during follow-upc** | | | | | | | | | | |
| 5d | M | 45 | 28.1 | Y | Bisoprolol | N | 40 | 8.8 | N | N |
| 6 | M | 48 | 23.1 | N | Atenolol | N | 39 | 11.1 | N | N |
| 7 | F | 58 | 19.1 | N | Bisoprolol | Y | 37 | 10.4 | N | Mild |
| 8 | M | 48 | 25 | N | Bisoprolol | Y | 30 | 6.4 | N | Mild |

a Defined as systolic blood pressure above 140mmHg

b Data at baseline is shown

c Data from the visit previous to the Afib event

d This patient had severe sleep apnea syndrome

Abbreviations: Afib: Atrial fibrillation, AoRR: Aortic root replacement, BB: Beta-blocker, BMI: Body mass index, HTA: Arterial hypertension, MVP: Mitral valve prolapse, MR: Mitral regurgitation, LAVi: left atrium volume index.
